# Modulation and Protection Effects of Antioxidant Compounds against Oxidant Induced Developmental Toxicity in Zebrafish

**DOI:** 10.3390/antiox9080721

**Published:** 2020-08-08

**Authors:** Nuria Boix, Elisabet Teixido, Ester Pique, Juan Maria Llobet, Jesus Gomez-Catalan

**Affiliations:** 1 Toxicology Unit, Pharmacology, Toxicology and Therapeutical Chemistry Department, Pharmacy School, University of Barcelona, Avda Joan XXIII s/n 08028 Barcelona, Spain; eteixido1511@ub.edu (E.T.); esterpique@ub.edu (E.P.); jmllobet@ub.edu (J.M.L.); jesusgomez@ub.edu (J.G.-C.); 2 INSA-UB Nutrition and Food Safety Research Institute, University of Barcelona, Food and Nutrition Torribera Campus, Prat de la Riba 171, 08921 Santa Coloma de Gramenet, Spain

**Keywords:** oxidative stress, zebrafish embryo, in vivo model, antioxidant effect

## Abstract

The antioxidant effect of compounds is regularly evaluated by in vitro assays that do not have the capability to predict in vivo protective activity or to determine their underlying mechanisms of action. The aim of this study was to develop an experimental system to evaluate the in vivo protective effects of different antioxidant compounds, based on the zebrafish embryo test. Zebrafish embryos were exposed to tert-butyl hydroperoxide (tBOOH), tetrachlorohydroquinone (TCHQ) and lipopolysaccharides from *Escherichia coli* (LPS), chemicals that are known inducers of oxidative stress in zebrafish. The developmental toxic effects (lethality or dysmorphogenesis) induced by these chemicals were modulated with n-acetyl l-cysteine and *N*ω-nitro l-arginine methyl ester hydrochloride, dimethyl maleate and dl-buthionine sulfoximine in order to validate the oxidant mechanism of oxidative stress inducers. The oxidant effects of tBOOH, TCHQ, and LPS were confirmed by the determination of significant differences in the comparison between the concentration–response curves of the oxidative stress inducers and of the modulators of antioxidant status. This concept was also applied to the study of the effects of well-known antioxidants, such as vitamin E, quercetin, and lipoic acid. Our results confirm the zebrafish model as an in vivo useful tool to test the protective effects of antioxidant compounds.

## 1. Introduction

Reactive oxygen species (ROS) and reactive nitrogen species (RNS) are products of cellular metabolism, which play a dual role in beneficial and deleterious effects over different organs [1]. Aerobic organisms have antioxidant defenses to protect cells from oxidative damage. These defenses can be enzymatic (antioxidant enzymes) or non-enzymatic (antioxidant compounds) [2]. The imbalance between reactive metabolite production and antioxidant defenses in the organism is denominated oxidative stress (OS) and can produce potential detrimental effects in the organisms [3]. The consequences of OS can be very variable depending on the reactive species implicated, the subcellular structure where they are generated, the organs or tissues implicated in the effect, the genetic characteristics of the organism or developmental stage, among other factors. It is a phenomenon which has been related to different processes (aging, cancer, diabetes, cardiovascular and neurodegenerative diseases, etc.) as it can damage and inhibit the normal function of lipids, proteins, and DNA [4].

Antioxidants are chemicals that can inhibit or prevent oxidation processes. Such compounds can be produced within the human body or absorbed from dietary intake [5]. The antioxidant capacity of compounds is usually evaluated by in vitro techniques as the oxygen radical absorbance capacity (ORAC) or the total radical-trapping antioxidant parameter (TRAP), which are useful for the high-throughput screening of the antioxidative or radical-scavenging capacities of the compounds [6]. There are also cell-culture approaches, such as the cell-based antioxidant assay (CAA), which uses Caco-2 cells that allow for the study of intracellular influence of antioxidant chemicals [7]. These in vitro assays and biological techniques, which are regularly used to evaluate antioxidant capacity, do not have predictive capability for the protective activity that natural compounds have in vivo, or to determine their underlying mechanisms of action [8]. In vivo assays of antioxidant capacity of natural compounds have been performed in mice [9], in rats [10], and using other animal models, such as *Caenorhabditis elegans* [11] and adult zebrafish [12]. However, until now none of these in vivo models have been established and validated to systematically evaluate the protective effects of natural compounds.

Zebrafish (*Danio rerio*, ZF) is a tropical fish of the *Cyprinidae* family. The ZF embryo is considered a potential tool for investigating environmental exposures with direct relation to human health [13]. The ZF embryo presents multiple advantages, from which it can be highlighted that it is an in vivo model which studies the whole organism, with the main characteristics of an in vitro model: easy maintenance, large number of offspring, rapid embryonic development, possibility to combine with other biochemical, cellular and molecular techniques, screening of compounds, application to high-throughput methods, etc. [14,15,16]. The ZF embryo has been used as a model to study alterations and diseases related to OS mechanisms: inflammation [17], senescence [18], teratogenicity [19], neurodegenerative [20], and cardiovascular diseases [21]. Furthermore, ZF presents antioxidant genes and enzymes to protect them against OS effects. These defenses are analogous to mammalian antioxidant systems [22,23]. The protective effects of some antioxidants against exposure to OS inducers in ZF embryos have been studied with the objective to investigate the antioxidant mechanisms of action and demonstrate the usefulness of these antioxidants against oxidative damage [19,24,25].

The aim of the present work was to design an experimental system based on the ZF embryo test, which could be the basis for the study of in vivo protective effects of chemicals with antioxidant activity against oxidant-induced developmental toxicity in ZF embryos.

## 2. Materials and Methods

### 2.1. Chemicals and Solution Preparation

Tetrachlorohydroquinone (TCHQ, CAS number 87-87-6), lipopolysaccharides from *Escherichia coli* 0111:B4 (LPS), *N*ω-nitro l-arginine methyl ester hydrochloride (L-NAME, CAS number 51298-62-5), DL-buthionine sulfoximine (BSO, CAS number 5072-26-4), (±)-α-tocopherol (vitamin E, CAS number 10191-41-0), (±)-α-lipoic acid (lipoic acid, CAS number 1077-28-7), and quercetin hydrate (quercetin, CAS number 337951) were obtained from Sigma-Aldrich, Madrid, Spain. Tert-butyl hydroperoxide (tBOOH, CAS number: 75-91-2) was acquired from TCI Europe and n-acetyl-l-cysteine (NAC, CAS number 616-91-1) and diethyl maleate (DEM, CAS number 141-05-9) were obtained from Cymit Química, Barcelona, Spain.

tBOOH, LPS, NAC, and DEM were directly dissolved in 0.3X Danieau’s buffer (17.4 mM NaCl; 0.23 mM KCl; 0.12 mM MgSO_4_·7 H_2_O; 0.18 mM Ca(NO_3_)_2_; 1.5 mM HEPES (N-(2-hydroxyethyl)piperazine-N′-(2-ethanesulfonic acid); pH 7.4). TCHQ, vit. E, quercetin, and lipoic acid were dissolved in 100% dimethyl sulfoxide (DMSO, Sigma-Aldrich, Madrid, Spain) and subsequently diluted in 0.3× Danieau’s buffer to a final DMSO concentration of 0.05 % (*v*/*v*).

Our previous experience with 0.05 % DMSO in 0.3× Danieau’s buffer clearly indicates that it does not produce any effects in lethality or dysmorphogenesis in ZF embryos, and it was not expected to modify the toxicity of the compounds. Moreover, DMSO is only expected to modify the permeability of chemicals if used at higher concentrations > 0.1% [26].

Concentrations of all chemicals are expressed in molarity, except for LPS that is given as µg/mL, due to the variable molecular mass of LPS, as it is part of the outer membrane of bacteria—in this case, *Escherichia coli*.

### 2.2. Animals and Embryo Production

Adult wild type ZF (BCN Piscicultura Iberica; Terrassa, Spain) were kept in aquariums with a closed flow-through system at 26 ± 1 °C and 10–14 h constant dark–light cycle. Females and males were housed separately and fed with commercial flakes and brine shrimp (Ocean Nutrition, San Diego, USA). The day before the experiments, females and males were transferred to a breeding tank (10 females; 8 males). ZF embryos were collected within 1 h after the onset of lights in the morning. They were extensively cleaned, and fertilized eggs were staged according to [27] and selected for subsequent exposure under a dissection stereomicroscope (Motic SMZ168, Motic China Group, LTD., Luwan, Shanghai, China). The study was approved by the Ethic Committee for Animal Experimentation of the University of Barcelona and by the Department of Environment and Housing of the Generalitat de Catalunya with license number DAAM 7971.

### 2.3. Exposure of Zebrafish Embryos to Oxidative Stress Related Compounds

To characterize the effects on embryonic development produced by compounds related to OS, ZF embryos were exposed to OS inducers, modulators of antioxidant status and antioxidants. For compounds which were dissolved in DMSO and diluted with Danieau’s buffer, a vehicle negative control group with 0.05% DMSO in 0.3× Danieau’s buffer was assayed.

Exposures to antioxidants and to modulators were performed from 2 to 26 h post-fertilization (hpf) in order to select, for each of the compounds of study, the highest concentration at which any effect in lethality or in embryonic development was observed (maximum tolerable concentration, MTC). From 26 to 50 hpf, embryos were incubated with 0.3× Danieau’s buffer with or without DMSO, depending on the dissolution of the compound of study.

Exposure to OS inducers was conducted from 26 to 50 hpf to select the working concentrations of these compounds for the experimental design. In this case, from 2 to 26 hpf, embryos were incubated with Danieau’s buffer with or without DMSO, depending on the dissolution of the compound of study.

Exposure of ZF embryos was semi-static and was carried out in 6-well plates (Greiner Bio-one, Frickenhausen, Germany). Ten embryos per group were selected and randomly distributed into the wells and filled with 5mL of the corresponding solution of the compound. Embryos were incubated at 26 ± 1 °C with a dark–light cycle of 10–14 h. Renewal of the medium and of the solutions was made every 24 h. Evaluation of the embryos was performed at different time points. Lethality was determined at 8, 26, and 50 hpf based on egg coagulation, the absence of tail detachment, or somite formation and the absence of heartbeat [28]. Dysmorphogenic effects were evaluated at 50 hpf by the total morphological score system described by [29]. For each compound of study, at least three independent experiments were performed using embryos from different spawning events (*n* = 3).

The percentage of lethality and of dysmorphogenesis was calculated per compound at every tested concentration, and the concentration–response curves for these effects were plotted. From these curves the concentration, which produced mortality to 50% of the embryos (lethal concentration 50, LC_50_), and the concentration at which 50% of the embryos presented at least one dysmorphogenic feature (effective concentration 50 for dysmorphogenesis, EC_50_), were calculated.

### 2.4. Pre-Exposure of the Embryos to Modulators of Antioxidant Status + Exposure to OS Inducers

To elucidate the OS role in the developmental effects produced by OS inducers, another assay was performed by modulating the ZF embryos’ OS responses through pre-exposure to the MTC of compounds which can affect OS conditions, and the posterior exposure to the working concentrations of OS inducers. NAC and L-NAME were used to potentiate antioxidant status, as NAC increases glutathione levels [30] and L-NAME inhibits nitric oxide production [31]. On the other hand, DEM and BSO were used to inhibit glutathione synthesis [32] by increasing the sensitivity of the embryos to OS.

At 2 hpf, embryos were pre-exposed to the MTC of modulators of antioxidant status for 24 h, and then washed with 0.3× Danieau’s buffer. At 26 hpf, embryos were exposed to OS inducers at the selected working concentrations. Lethality and dysmorphogenesis were evaluated as previously described, and concentration–response curves were plotted. A comparison of the concentration–response curves of pre-exposure to modulators of antioxidant status + exposure to OS inducers with concentration–response curves of OS inducers exposure was performed.

### 2.5. Pre-Exposure of the Embryos to Antioxidant Compounds + Exposure to OS Inducer

To detect the protective effects of chemicals against oxidant induced developmental toxicity in ZF embryos, different compounds with well determined antioxidant activity were assayed.

A pre-exposure to the MTC of vitamin (vit.) E, lipoic acid and quercetin was performed from 2 to 26 hpf, followed by a washing step with 0.3× Danieau’s solution and the exposure to the working concentrations of the selected OS inducer for 24 h. Evaluation of the embryos was performed as described before, and concentration–response curves were graphically represented. A comparison between the concentration–response curves of pre-exposure to antioxidants + exposure to the selected OS inducer with the concentration–response curve of the exposure to the selected OS inducer was performed.

### 2.6. Data Evaluation

Comparison of categorical variables was performed with the Fisher’s exact test. Concentration–response curves for lethality and dysmorphogenesis were fitted to all the data using the Hill model in GraphPad Prism 6 software and compared with the extra sum-of-squares F test, which compares the parameters fit to datasets (GraphPad Software, La Jolla, CA, USA). Confidence intervals were set at 95% and a probability of *p* < 0.05 was considered as statistically significant.

## 3. Results

### 3.1. Characterization of the Effects of Oxidative Stress Related Compounds in Zebrafish Embryos

The results of the characterization of the lethal and dysmorphogenic effects produced by ZF embryos exposure to OS inducers, modulators and antioxidants are shown in Table 1.

OS inducers produced developmental effects in zebrafish embryos (lethality and dysmorphogenic effects), which were concentration-dependent. Modulators of antioxidant status and antioxidants did not produce lethality at the studied concentrations, and the dysmorphogenic effects observed in the embryos exposed to the tested compounds were mainly developmental delay, cardiac oedema, and brain necrosis, which were not specific alterations. The only compound-specific effect was observed in TCHQ exposure, which produced an effect in the pigmentation of the embryos.

### 3.2. Pre-Exposure to Modulators of Antioxidant Status + Exposure to OS Inducers

We attempted to modulate the embryotoxic and lethal effects produced by OS inducers in zebrafish embryos by pre-exposing them to a set of known modulators of antioxidant status in zebrafish (Table 2), in order to evaluate if the effects produced by OS inducers were caused by an OS mechanism.

**Figure 1:**
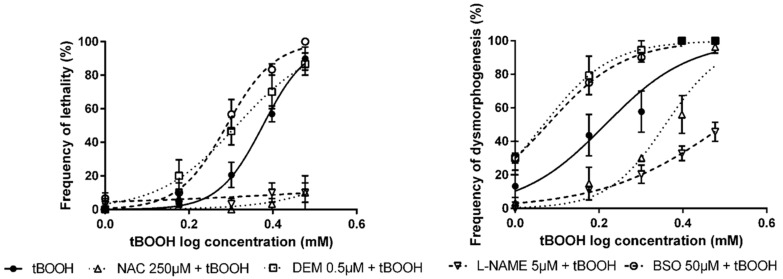
Concetration–response curves for lethality and dysmorphogenesis of tert-butyl hydroperoxide (tBOOH) alone or in combination with modulators of antioxidant status.

In embryos which were exposed to tBOOH, a pre-exposure to NAC and L-NAME significantly drifted the tBOOH concentration–response curves to higher concentrations (Figure 1), the fact that, at the NAC and L-NAME pre-exposure group, no significant effects in mortality of the embryos were observed being of special importance. On the contrary, when ZF embryos where pre-exposed to DEM and BSO, a significant shift in the concentration–response curves to lower concentrations of tBOOH was observed (Figure 1). As described before, the tBOOH concentration–response curve was generated after a pre-incubation of the embryos for 24 h in 0.3× Danieau’s buffer without DMSO, due to the lack of effects of DMSO in ZF development.

**Figure 2:**
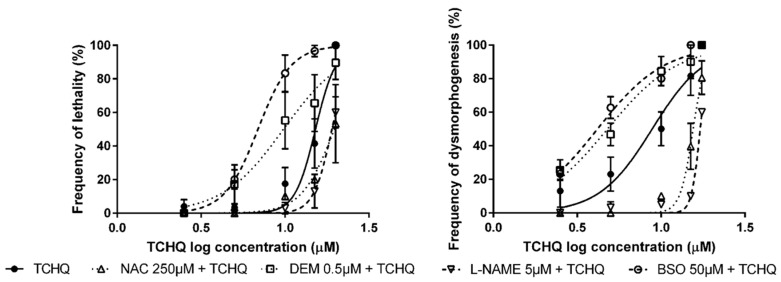
Concentration–response curves for lethality and dysmorphogenesis of tetrachlorohydroquinone (TCHQ) alone or in combination with modulators of antioxidant status.

The modulation of antioxidant status in embryos exposed to TCHQ presented similar results to tBOOH. When ZF embryos were pre-exposed to NAC and L-NAME, the concentration–response curves for lethality and dysmorphogenesis were significantly shifted to higher concentrations of TCHQ (Figure 2). On the other hand, assays conducted with pre-exposure to DEM and BSO produced a statistically significant drift in the concentration–effect curves for lethality and dysmorphogenesis to lower concentrations of TCHQ (Figure 2).

**Figure 3:**
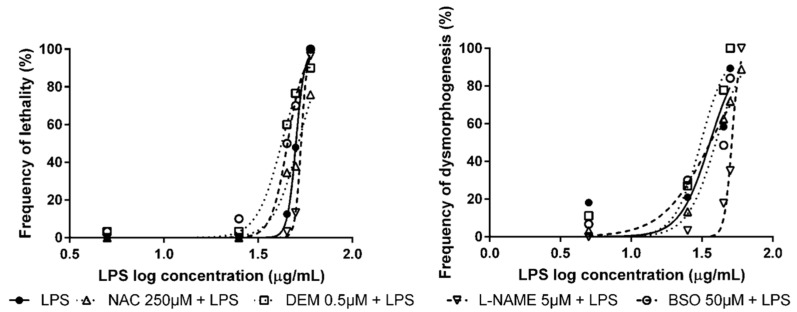
Concentration–response curves for lethality and dysmorphogenesis of lipopolysaccharides of *Escherichia coli* 0111:B4 (LPS) alone or in combination with modulators of antioxidant status.

Pre-exposure of ZF embryos to NAC, DEM, L-NAME, and BSO followed by LPS exposure at the selected working concentrations shifted the lethality concentration–effect curves significantly. In the analysis of dysmorphogenic effects in ZF embryos, no significant effects were observed in embryos pre-exposed to NAC, DEM, and BSO, and subsequently exposed to LPS. Only a significant reduction in dysmorphogenic effects was observed in L-NAME pre-exposed embryos (Figure 3). As described in the previous section, embryos were pre-incubated with 0.3× Danieau’s buffer without DMSO, followed by LPS exposure and calculation of the concentration–response curve, due to the lack of effects of DMSO in ZF development.

The modulation of antioxidant status in embryos exposed to tBOOH produced more consistent results than other OS inducers. The observed effects in the embryonic development were general alterations not compound-specific. tBOOH was selected as the general OS inducer for the study of protective effects of antioxidant compounds.

### 3.3. Detection of Protective Effects of Antioxidant Compounds in Zebrafish Embryos

The second part of the study consisted in the use of tBOOH as a general OS inducer for the detection of compounds with very well-known antioxidant capacity. ZF embryos were exposed from 2 to 26 hpf to antioxidant compounds (vit. E, lipoic acid, and quercetin), before exposing them to tBOOH from 26 to 50 hpf.

**Figure 4:**
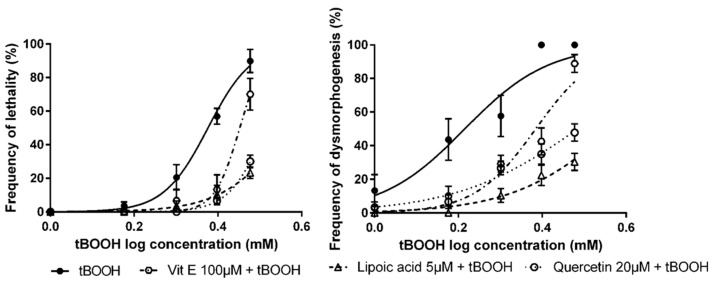
Concentration–response curves for lethality and dysmorphogenesis of tBOOH alone or in combination with different antioxidant compounds.

In all cases, pre-exposure to the studied compounds produced a significant drift in the concentration–response curves of lethality and dysmorphogenesis to higher concentrations of tBOOH (Figure 4), which may indicate an antioxidant effect.

The LC_50,_ after tBOOH exposure, was 2.38mM, and values obtained after vit. E, lipoic acid, and quercetin exposure were 2.83 mM, 3.72 mM, and 3.26 mM, respectively. For the EC_50_ values, the situation was similar, from tBOOH exposure, the EC_50_ was 1.64 mM, while pre-exposure to the studied compounds returned an EC_50_ of 2.42 mM for vit. E, 3.70 mM for lipoic acid and 3.05 mM for quercetin (Table 3).

## 4. Discussion

Oxygen is an essential element for cell life and, from its metabolism, some toxic derivatives are produced, such as ROS, which are highly reactive to biological molecules and can produce OS [33]. An important factor that could prevent OS effects is the alimentary antioxidants intake. For this reason, the study of antioxidant capacity of compounds has been gaining interest in the past few years. It has been postulated that, in order to evaluate the antioxidant potential, a method which includes in vivo techniques would have more impact on the results because OS implies mechanisms which depend on many system conditions, especially the kinetic part of the reactions [34]. We have proposed the ZF embryo test, which could be a valuable in vivo method to test the antioxidant capacity of compounds, with the main advantages of an in vitro technique.

In the first part of this study, we characterized the embryotoxic and dysmorphogenic effects of several compounds, which have an OS-related mechanism of action on the ZF embryos: tBOOH, TCHQ, and LPS. The induction of OS by tBOOH is due to its capacity to generate butoxyl radicals which deplete antioxidant systems and lead to cell death [35], and it has been previously used in ZF embryos to induce OS [36]. TCHQ can induce OS by producing superoxide radicals, favoring the depletion of the reduced glutathione concentrations [37], and it has also been observed that TCHQ can produce DNA strand breakage in cells [38]. LPS is a microbial product of bacteria and its contribution to ROS production has been studied as a secondary effect to inflammation [39]. It has been used as an OS inducer in different in vitro and in vivo models [40]. All the studied OS inducers produced a significant increase in lethality and in the production of dysmorphogenesis in the exposed ZF embryos.

In order to check if the observed effects in ZF embryos could be produced by an OS mechanism, we performed assays of modulation of the embryos’ antioxidants statuses with compounds related to OS. The modulation was carried out through raising or decreasing the antioxidant defenses of the embryos with NAC and L-NAME, and DEM and BSO, respectively. NAC is an antioxidant compound, which is a rate-limiting substrate in glutathione synthesis, and it can also act as a scavenger of free radicals [41]. L-NAME is an inhibitor of nitric oxide synthase, the enzyme responsible for nitric oxide synthesis. As a result of this inhibition, it reduces the production of endogenous nitric oxide, which is a compound that can produce reactive nitrogen species and consequently, OS [30]. DEM is an alkylating agent that can produce a conjugation and depletion of glutathione [42], and it can also activate the nuclear factor (erythroid-derived 2)-like 2 (Nrf2) pathway [22], and BSO is an antioxidant molecule suppressor, which specifically inhibits γ-glutamyl cysteine synthetase, the enzyme for glutathione biosynthesis, and causes the depletion of glutathione levels [43].

In general terms, we have demonstrated that the lethal and dysmorphogenic effects of tBOOH and TCHQ were significantly reduced when the embryos were pre-treated with antioxidant compounds (NAC and L-NAME). From the opposite position, the observed effects in mortality and in dysmorphogenesis were significantly increased when ZF embryos were pre-exposed to compounds which decrease the antioxidant status (DEM and BSO). We could conclude that tBOOH and TCHQ produced their embryolethal and dysmorphogenic effects in ZF embryos by an OS mechanism of action. No significant effects in dysmorphogenesis related to OS were observed in the LPS treatment group. The observed effects in the lethality of ZF embryos exposed to LPS could be more related to its mechanism as an inflammation inducer [44] than as an OS mechanism. Nevertheless, the effects of these compounds, associated with an OS mechanism, should be verified by analyzing parameters directly related to OS, like evaluation of the expression of OS-related genes in the exposed ZF embryos.

Because of its consistent results, tBOOH was selected as the OS inducer to be used to evaluate the antioxidant potential of compounds. To validate the use of tBOOH to detect the protective effects of antioxidant compounds, ZF embryos were pre-exposed to diverse compounds with a well-established antioxidant capacity (vit. E, lipoic acid, and quercetin) and posteriorly exposed to tBOOH. In addition, a statistical analysis was performed by comparing the concentration–effect curves for lethality and for dysmorphogenesis obtained in both experiments: tBOOH alone and antioxidants + tBOOH.

Vit. E is a compound with free-radical scavenging activity, which leads to an antioxidant action that has been demonstrated in vitro [45]. Lipoic acid is a thiol regenerating compound, which increases the level of glutathione. It inhibits the formation of hydroxyl radicals, and it also scavenges ROS [46]. Quercetin is a flavonol found in apples, tea, and onions, and exerts its antioxidant effect through different bioactive effects. Its main antioxidant mechanism of action is through quenching different radicals, such as hydroxyl, peroxyl, and superoxide, as well as nitric oxide and lipid oxidation [5]. Quercetin can induce antioxidant gene expression through the activation of Nrf2 [47]. Among these, quercetin can also modulate mitochondrial biogenesis by reducing ROS production in various cell types [48]. The pre-exposure of the embryos to vit. E, quercetin, and lipoic acid, followed by the exposure to the OS inducer, has confirmed the protective effects of well-known antioxidant compounds against oxidant-induced developmental toxicity in ZF. In all the cases, the pre-exposure of ZF embryos to the compounds followed by the exposure to the selected concentrations of tBOOH produced a significant shift of the concentration–effect curves of lethality and dysmorphogenesis. These results indicated the preventive effect of vit. E, lipoic acid, and quercetin against the toxic effects of tBOOH, which were related to an OS mechanism of action. The antioxidant effect of these compounds versus oxidant effects produced by the OS inducer should be confirmed by the application of antioxidant capacity evaluation methods.

The ZF embryo test has been widely used to study different types of compounds, including OS-related chemicals. Recently, a new stable transgenic line has been developed for the rapid detection of oxidative stress, although it has not been systematically tested to evaluate the antioxidant capacity of chemicals [49]. The results of our study are similar to those observed by the authors in [25], in which they observed the protective effect of vit. E in ZF embryos exposed to PCB126, which causes OS. There are other studies in which they evaluated the effects of compounds, which may have part of its mechanism of action related to oxidative injury, such as ethanol, in ZF embryos [19]. In this case, they analyzed and confirmed the partial prevention of ethanol-induced cardiovascular disfunction by lipoic acid in ZF embryos. Natural antioxidant compounds, such as quercetin, have demonstrated their antioxidant capacity and their protective activity against different diseases using the ZF embryo test [12], reinforcing the results obtained in our study.

## 5. Conclusions

The ZF embryo has been established as the basis for the study of the modulative and protective effects of antioxidant compounds in oxidant induced developmental toxicity in ZF. An experimental design using tBOOH as an OS inducer has been developed in the present study. The evaluation of the OS-related effects produced by tBOOH was estimated by a modulation of the antioxidant status assay with NAC, L-NAME, DEM, and BSO. The study of the protective effects of antioxidant compounds was performed with pre-exposure of ZF embryos to vit. E, lipoic acid, and quercetin, which are compounds with a well-established antioxidant capacity, and the protective effect of these compounds on developmental effects in the embryos was confirmed.

Our experimental system could be used as a valuable in vivo tool for testing compounds with presumable antioxidant activity, with advantages in respect to other techniques used in the evaluation of the antioxidant capacity (analytical or cell-based assays).

Further studies should be done to extensively characterize the effects of tBOOH as an OS inducer, as well as to evaluate the antioxidant capacity of compounds, in order to establish an OS model based on ZF embryos to study new antioxidant compounds and the mechanism of action by which they exert their antioxidant activity.

## Figures and Tables

**Table 1:** Characterization of lethality and dysmorphogenesis in zebrafish embryos, produced by oxidative stress-related compounds.

Compounds	Range of Concentrations	MTC	LC_50_	EC_50_	Exposure Window
*OS Inducers*					
Tert-butyl hydroperoxide (tBOOH)	1–4 mM	n.d.^a^	2.4 mM	1.6 mM	26–50 hpf
Tetrachlorohydroquinone (TCHQ)	2.5–20 µM	n.d.^a^	16.0 µM	3.9 µM	26–50 hpf
Lipopolysaccharides from *Escherichia coli* 0111:B4 (LPS)	5–60 µg/mL	25 µg/mL	50.1 µg/mL	35.9 µg/mL	26–50 hpf
*Modulators of Antioxidant Status*					
n-acetyl-l-cysteine (NAC)	50–2500 µM	250 µM	1874 µM	920.6 µM	2–26 hpf
Diethyl maleate (DEM)	0.1–100 µM	0.5 µM	n.d.^b^	1.5 µM	2–26 hpf
*N*_ω_-nitro l-arginine methyl ester hydrochloride (L-NAME)	0.1–100 µM	5 µM	n.d.^c^	44.36 µM	2–26 hpf
DL-buthionine sulfoximine (BSO)	1–5000 µM	50 µM	n.d. ^c^	2722 µM	2–26 hpf
*Antioxidants*					
Vit. E	1–1000 µM	100 µM	n.d.^d^	n.d.^d^	2–26 hpf
Lipoic acid	0.1–1000 µM	5 µM	116.4 µM	n.d.^c^	2–26 hpf
Quercetin	0.1–30 µM ^e^	20 µM	n.d.^d^	n.d.^d^	2–26 hpf

Range of tested concentrations, maximum tolerable concentration (MTC), lethal concentration 50 (LC_50_), effective concentration 50 for dysmorphogenesis (EC_50_) and exposure window for each of the studied compounds. n.d.: data were not determined. ^a^: MTC was not determined because the compound produced lethal or dysmorphogenic effects at all the studied concentrations. ^b^: LC_50_ was not calculated because no lethal effects were observed until the highest concentration, where lethality was of 100%. ^c^: LC_50_ or EC_50_ was not calculated because no significant effects in the mortality of the embryos were observed. ^d^: LC_50_ and EC_50_ were not calculated because the compounds did not produce lethal or dysmorphogenic effects at any of the tested concentrations. ^e^: Quercetin solution precipitated from 30 µM. It was not possible to evaluate the effects at higher concentrations.

**Table 2:** Lethality and dysmorphogenesis effective concentration values in zebrafish embryos on the modulation of developmental effects produced by OS inducers.

Modulator of Antioxidant Status	OS Inducer	LC_50_ (95% CI)	EC_50_ (95% CI)
*None ^1^*	Tert-butyl hydroperoxide (tBOOH)	2.38 mM (2.28–2.48)	1.64 mM (1.44–1.87)
n-acetyl-l-cysteine (NAC)		n.d.	2.28 mM ** (2.11–2.46)
*N*_ω_-nitro l-arginine methyl ester hydrochloride (L-NAME)		n.d.	3.17 mM *** (2.85–3.52)
Diethyl maleate (DEM)		2.06 mM * (1.78–2.38)	1.17 mM ** (1.07–1.29)
DL-buthionine sulfoximine (BSO)		1.95 mM *** (1.85–2.05)	1.20 mM * (1.07–1.33)
*None*	Tetrachlorohydroquinone (TCHQ)	15.2 µM (13.8–16.7)	8.84 µM (7.15–10.9)
NAC		19.6 µM * (16.6–23.3)	15.5 µM *** (14.8-16.3)
L-NAME		19.0 µM * (17.3–20.9)	17.1 µM *** (16.9–17.3)
DEM		9.78 µM ** (7.31–13.1)	4.79 µM * (3.88–5.91)
BSO		6.89 µM *** (6.13–7.75)	4.17 µM ** (3.62–4.81)
*None ^1^*	Lipopolysaccharides from *Escherichia coli* 0111:B4 (LPS)	50.1 µg/mL (48.6–51.8)	36.0 µg/mL (28.4–45.6)
NAC		51.6 µg/mL * (48.8–54.5)	39.6 µg/mL (35.0–44.8)
L-NAME		53.4 µg/mL * (51.9–55.0)	51.3 µg/mL ** (49.6–53.0)
DEM		42.1 µg/mL *** (37.9–46.8)	31.1 µg/mL (26.3–36.6)
BSO		45.2 µg/mL ** (43.2–47.4)	36.2 µg/mL (29.4–44.5)

Lethal concentration 50 (LC_50_), effective concentration 50 for dysmorphogenesis (EC_50_) and 95% confidence interval. Statistically significant differences with respect to the group, which was not exposed to any modulator: *: *p* < 0.05; **: *p* < 0.01; ***: *p* < 0.001; n.d.: no lethality was observed; ^1^ A unique tBOOH and LPS concentration–response curve was generated with the dissolution of the compounds in Danieau’s buffer without DMSO and compared to all the concentration–response curves of the groups pre-exposed to chemicals (initially dissolved or not in DMSO) due to the lack of effect of DMSO in the embryonic development of ZF.

**Table 3:** Effects of antioxidant compounds in lethality and dysmorphogenesis of zebrafish embryos exposed to tBOOH.

Antioxidant Compounds	OS Inducer	LC_50_ (95% CI)	EC_50_ (95% CI)
*None ^1^*	Tert-butyl hydroperoxide (tBOOH)	2.38 mM (2.28–2.48)	1.64 mM (1.44–1.87)
Vitamin E		2.83 mM *** (2.70–2.69)	2.42 mM *** (2.17–2.70)
Lipoic acid		3.72 mM *** (3.14–4.40)	3.70 mM *** (3.03–4.51)
Quercetin		3.26 mM *** (2.83–3.76)	3.05 mM *** (2.64–3.54)

Lethal concentration 50 (LC_50_), effective concentration 50 for dysmorphogenesis (EC_50_) and 95% confidence interval. Statistically significant differences with respect to the group which was not exposed to any antioxidant compound: ***: *p* < 0.001; ^1^ A unique tBOOH concentration–response curve was generated and compared to all the concentration–response curves of the antioxidants pre-exposure groups (initially dissolved or not in DMSO) due to the lack of effect of DMSO in the embryonic development of ZF.
